# Cultural competence in critical care nurses and its relationships with empathy, job conflict, and work engagement: a cross-sectional descriptive study

**DOI:** 10.1186/s12912-023-01285-x

**Published:** 2023-04-12

**Authors:** Mohsen Soleimani, Sajad Yarahmadi

**Affiliations:** 1 Nursing Care Research Center, Semnan University of Medical Sciences, Semnan, Iran; 2 Student Research Committee, Semnan University of Medical Sciences, Semnan, Iran; 3 Social Determinant of Health Research Center, School of Nursing & Midwifery, Lorestan University of Medical Sciences, Khorramabad, Iran

**Keywords:** Cultural competence, Critical care nurse, Empathy, Job conflict, Work Engagement

## Abstract

**Background:**

Cultural competence is more important than ever for nurses today; therefore, it may be helpful to learn more about it and examine how it relates to empathy, job conflict, and work engagement. The purpose of this study was to determine (a) the level of cultural competence, empathy, job conflict, and work engagement; (b) the relationship between cultural competence, demographic information, and main variables; (c) the predictors of cultural competence among critical care nurses.

**Methods:**

A multicenter, descriptive cross-sectional study was conducted in Iran from August to October 2022. Through convenience sampling, 153 critical care nurses from three hospitals participated. The research tool consisted of five parts: Demographic information questionnaire, Cultural Competence Questionnaire, Jefferson Scale Empathy, Dobrin Job Conflict, and Utrecht Work Engagement, which were collected by paper self-report. Descriptive statistics, the correlation between variables, and linear regression were used to analyze the data.

**Results:**

Among critical care nurses (response rate 79.27%), the mean (SD) scores for cultural competence, empathy, job conflict, and work engagement were 74.05 (7.96), 83.44 (29.17), 11.00 (2.38), and 43.69 (16.33), respectively. There was a significant correlation between cultural competence and age (r = 0.46, p = 0.001), marital status (r = 0.27, p = 0.004), academic degree (r = 0.44, p = 0.001), work experiences (r = 0.43, p = 0.001), empathy (r = 0.50, p = 0.001), and job conflict (r=-0.16, p = 0.049). Academic degree (β = 0.36, p < 0.001) and empathy (β = 0.26, p < 0.001) were significant explanatory variables that predict cultural competence.

**Conclusion:**

In Iranian critical care nurses, cultural competence and job conflict were moderate, empathy was good, and work engagement was poor. There was a significant relationship between cultural competence, age, marital status, academic degree, work experiences, empathy, and job conflict. Academic degree and empathy predict cultural competence.

## Background

### Culture in Iran

Culture can affect most aspects of human life and influence people’s behaviors, beliefs, and values [1]. Iranian society is the cradle of various cultures; local and foreign cultures live there, and it has a rich historical framework that is culturally, linguistically, ethnically, and religiously diverse [2, 3]. In addition, there is a rise in intercultural diversity due to immigration from the two surrounding nations of Iraq and Afghanistan and Iran’s flourishing medical tourism industry [3]. Increased cultural diversity is considered a severe challenge to healthcare systems, and these systems should provide high-quality care to patients with different needs and cultural backgrounds [4]. Nurses, as the largest group of healthcare workers, need to consider patients’ cultures as a component of health-focused care and should be able to communicate effectively with patients from diverse cultures [3]. The study findings from Iranian nurses’ experience caring for patients from other cultures showed that minorities and small cultures were neglected, and hospitalization of such people in hospitals and other clinics was unclear [2].

### Iran’s health and nursing education system

Iran, with a population of 80 million, is one of the most populous countries in West Asia, and three pillars of Iranian healthcare are the public-governmental system, the commercial sector, and non-governmental groups [5]. The country spent 6.7% of its gross domestic product on health in 2019, and almost 90% of Iranians have health insurance [6]. The Iranian nursing education system is crucial to achieving the objectives of the healthcare system, and in comparison to other developed and developing nations, it does not hold an unsuitable position [7]. Iran’s nursing education includes three programs bachelor, master, and doctoral. There are different nursing master’s fields in Iran, including critical care nursing, medical-surgical nursing, pediatric nursing, psychiatric nursing, neonatal intensive care nursing, community health nursing, military nursing, rehabilitation nursing, geriatric nursing, emergency nursing, nursing management, pediatric critical care nursing [7].

### Cultural competence

Mobaraki et al. define cultural competence as “a dynamic process that can be generalized and taught that improves over time with more experience and leads to effective communication with people from other cultures” [1]. To care for people of different religions, ethnic groups, countries, and races, we do not need to know each culture’s social practices, dominant beliefs, or rules; instead, in moments of vulnerability and fear, we should treat patients how we want to be treated [8]. A patient-centered approach and exhibiting respect, sensitivity, composure, partnership, honesty, understanding, curiosity, tolerance, and a positive attitude toward them are the keys to developing cultural competence that can improve patient outcomes [8, 9]. The results of previous studies in Iran show that cultural competence is moderate in general nurses [10], weak in undergraduate students [11] and critical care nurses (CCNs) [12].

### Cultural competence in CCNs

The CCNs are team workers with the training, experience, and knowledge necessary to provide critical care units and care for patients in complex conditions [13, 14]. American association of colleges of nursing defines “progressive and critical care nursing as a specialty that manages the human responses to actual or potentially life-threatening problems” [13]. Culturally sensitive includes understanding one’s culture, open and sensitive communication, and collaborating strategies with the patient and family [15]. Providing culturally sensitive nursing care is essential; nevertheless, it can be more difficult in the critical care unit than in other clinical settings. It is only possible for CCNs to deliver high-quality care for patients from diverse cultural backgrounds if they can demonstrate some fundamental cultural competence [16]. Here, the patient’s situation is complex and vulnerable, family members are involved, and the nature of the nursing activity calls for a high level of cultural competence [14]. Unawareness of cultural differences can lead to misunderstandings, reduce patient satisfaction, and even result in nursing errors [16].

The World Federation of Critical Care Nurses published a policy paper on culturally sensitive; It emphasized the need for CCNs to have the knowledge, skills, and qualities necessary to provide culturally sensitive care [17]. Nurses’ cultural competence can significantly affect health system efficiency worldwide [3]. Almutairi et al.‘s study showed that nurses’ country of birth might influence their perceptions of cultural competence. Also, this study showed that cultural competence requires exposure to caring for patients from various cultures and countries and is associated with cultural knowledge and awareness [18]. Iran is one of the countries exporting nurses to Anglo-Saxon countries (US, UK, Australia, NZ & Canada) [19]. According to our field observations, since the CCNs in Iran have higher expertise than others, they are more welcome to work in Anglo-Saxon countries. Despite the importance of cultural competence for Iranian CCNs, more is needed to know how to improve it.

### Empathy

Empathy is a crucial component influencing therapeutic relationships and the capability to comprehend the patients’ feelings, worries, and perspectives [20]. The nurse-patient interaction is built on empathy, which is seen as a core ability for medical professionals and is essential for the desired patient outcomes [20, 21]. Stavropoulou et al.‘s study showed that empathy in the critical care unit was perceived as closely related to patients’ outcomes and quality of care [22]. Despite the importance of this issue, the results of Gahaedi et al.‘s study showed that the mean level of empathy in nurses working in ICUs was lower than those in other departments [23]. However, another study in Jordan found that CCNs had high levels of empathy [24]. In Bernhard et al.‘s study, cultural emotions/empathy was recognized as one of the healthcare professionals’ components of cultural competence [25]. Barzykowski et al.‘s study showed that Polish health professionals have a significant relationship between intercultural competence and empathic sensitivity [26]. Also, Sohrabi et al. showed that cultural competence training enhances learners’ empathy in clinical settings [27]. Cultural competence can be the cornerstone of the two sides to build empathy dialogue and are intertwined with empathy and reinforce each other [20]. The previous studies confirmed that nurses’ cultural competence and empathy have a positive correlation [20, 28]. The relationship between cultural competence and empathy in CCNs must be investigated despite this literature.

### Job conflict

Conflict on the job is an undesirable dynamic process between parties that arises from perceived disagreements and interference with the parties’ goals and results in adverse emotional reactions [29]. Interpersonal conflict is a common workplace feature for nurses, and conflict among nurses is a persistent problem [29, 30]. The level of conflict between Iranian CCNs has been reported as moderately high [30]. Abd El-Moneim Ahmed and Gaballah’s study showed that more than half of Egyptian CCNs had moderate or high conflict [31]. Conflicts are related to nursing consequences, such as the quality of nursing care [32]. Conflict management is associated with better nurse performance, which could ultimately improve patient care in critical care units [33]. A study showed that intercultural competence was related to conflict resolution strategies [34]. Job conflicts management and promoting cultural competence can effectively improve the quality of care [32, 35, 36]. Based on our hypotheses, job conflict can be a negative factor in promoting cultural competence. Our searches did not find any studies showing the relationship between job conflict and cultural competence, so it seems necessary to determine the relationship between them. Also, It can strengthen the existing literature on cultural competence and be helpful for future studies.

### Work engagement

Work engagement is a pleasant, contented mental state associated with vitality, dedication, and absorption. For nurses to be engaged at work, there needs to be both trust and autonomy [37, 38]. Higher stages of personal initiative, lower hospital mortality rates, and much higher financial profitability of organizations can all result from nurses feeling engaged at work [37]. Van Mol et al. demonstrated that work engagement in CCNs was correlated with agreeableness, conscientiousness, and emotional stability; they also showed that work engagement balanced responses to work-related stress [39]. The study’s results by Kim et al. showed that work engagement could raise nursing services’ quality by encouraging nurses to use their full range of competence and expertise [38]. Since work engagement affects the competence of nurses, it may also be related to cultural competence.

According to this literature review and our hypotheses, the current study aimed to determine; (a) the levels of cultural competence, empathy, job conflict, and work engagement; (b) the relationship between cultural competence, demographic information, and main variables; (c) the predictors of cultural competence in CCNs.

## Methods

### Design

A multicenter, descriptive cross-sectional study was conducted from August to October 2022.

### Setting

The study settings were 14 critical care units, including eight ICUs, four CCUs, and two dialysis wards selected from three educational hospitals affiliated with Lorestan University of Medical Sciences, Khorramabad, Iran. Khorramabad is the capital of Lorestan province, located in the southwest of Iran.

Critical care units are a subset of ICUs, CCUs, and dialysis wards in Iranian hospitals’ organizational charts. Nursing students complete critical care courses in these settings, per the nursing curricula. Most of the CCNs in Iran have a bachelor’s degree [40].

### Participants

Participants comprised nurses working in critical care units. The following criteria were required for inclusion: nurses with a bachelor’s degree or higher who were employed in the critical care units and have at least six months of work experience there. An exclusion criterion was unwilling to participate in the study, failing to respond to at least one of the five questionnaires.

### Sample size

Based on a related study, the Pearson correlation coefficient between cultural competence and empathy was calculated at r = 0.3 [20]; the sample size was computed using the following formula (n = 102). The final sample size was 153 people after multiplying this number by 1.5 to account for the design impact and specifying a power of 90%, α = 0.05, β = 0.1, Z (1-α / 2) = 1.96, and Z (1-β) = 1.28.

$$\frac{{{(Z}_{1-\alpha /2}+{Z}_{1-\beta })}^{2}}{({\frac{1}{2} ln\frac{1+r}{1-r})}^{2}}$$

### Sampling

A convenience sampling technique was used to select the participants. A total of 197 CCNs met the inclusion criteria. Sampling was stopped when we reached the calculated sample size.

### Research tools

The research tool comprised five questionnaires, including demographic data, cultural competence, empathy, job conflict, and work engagement. Demographic data included age, gender, marital status, academic degree, and length of work experience.

### Cultural competence questionnaire

To develop a native theoretical model of cultural competence, Mobaraki et al. (2019) conducted qualitative research using the grounded theory approach. The initial tool was designed after a qualitative study and searching similar studies. Then, the tool validity was assessed by evaluating the face and content validity and performing surveys and psychometrics. Finally, the data were statistically analyzed through exploratory factor analysis. The tool’s dimensions were based on the theoretical model, and the initial items were then extracted. The final 25-item questionnaire was developed for the Iranian population in five areas of theoretical and practical learning, clinical application, cultural skill, cultural excellence, and cultural competence. A five-point Likert scale was used for scoring (1 = rarely, 5 = almost always). These categories for the score range from 93 to 100 (very strong), 81–92 (strong), 80 − 64 (moderate), 44–63 (weak), and 20–43 (very weak). This questionnaire has a Cronbach’s alpha coefficient of 0.91 and an ICC of 0.93 [1].

### Jefferson Scale Empathy

This 20-item scale of empathy for healthcare professionals has been psychometrically validated. A seven-point Likert scale was used for scoring (totally disagree = 1, totally agree = 7). The maximum and minimum scores were 20 and 140, respectively. Higher scores indicate more empathetic care behaviors with the patients. The Persian version of this scale has good face and content validity, Cronbach’s alpha coefficient is 0.83, and its ICC is 0.82 [41].

### Dobrin Job Conflict

This scale consists of 20 questions with two options (totally disagree = 0, totally agree = 1) and mainly scored on positive and negative responses. The ratings ranged from 0 to 20, respectively. The score categorization would be designed as 15–20 (high), 4–14 (moderate), and 0–3 (low). Hosseini et al. (2012) validated it for the Iranian population. This scale’s Cronbach’s alpha coefficient is 0.95 [42].

### Utrecht Work Engagement

This scale consists of 17 items, and scoring was done using a five-point Likert scale (1 = rarely, 5 = almost always). The scores ranged from zero to 102, respectively. Higher scores indicate a higher level of work engagement. Torabinia et al. (2017) validated the Persian version to measure work engagement in Iranian nurses and other medical professionals. This scale’s Cronbach’s alpha coefficient is 0.84, and the ICC is 0.91 [43].

### Procedure

Following approval from the Semnan and Lorestan University of Medical Sciences, the data were collected using paper self-report questionnaires. The researcher briefed the nurses on the purposes of the study and what was covered in the questionnaires. The participants who agreed to participate in the study provided written consent. Afterward, the questionnaires were distributed to the participants.

### Statistical analyses

Data on the general characteristics and responses of the participants related to cultural competence, empathy, job conflict, and work engagement were summarized using descriptive statistics. Mean comparison techniques were used to assess the variations in cultural competence according to the participant’s demographic variables. The Spearman (i.e., ordinal scale or non-normally distributed variables), Pearson (i.e., normally and linear distributed variables) correlation tests, intragroup correlation coefficient, and linear regression analyses were used to assess the study hypotheses in the inferential section. Before performing the regression analysis, we confirmed that our data satisfied the basic regression assumptions, such as homogeneity of variance and multi-collinearity. We utilized imputation for the missing data (mean substitution). The alpha error level was set to a maximum of 0.05. In order to analyze the data, IBM SPSS Statistics 22.0 was used [44].

## Results

A total of 153 CCNs from three hospitals participated in this study (response rate 79.27%). Kolmogorov-Smirnov and Shapiro–Wilk tests showed that all main variables had a normal distribution (p > 0.05).

### Demographic information and level of the main variables

Participants’ mean (SD) age was 31.33 (6.52) years. The results showed that most participants were women (75.20%) and married (62.70%). Also, most participants had bachelor’s academic degrees (81.70%), and others had master’s degrees in nursing (18.30%). The mean (SD) work experience was 9.04 (6.63) years. Additional personal and professional data are included in Table 1. Also, Table 2 shows the mean (SD) of CCNs’ cultural competence, empathy, job conflict, and work engagement.

**Table 1:** Description and correlation of cultural competence and demographic variables (n = 153)

Variables	N (%)	Cultural Competence Mean (SD)	r (p-value)
Age (years) 22–29 30–39 ≥ 40	72 (47.10) 63 (41.20) 18 (11.80)	69.82 (7.29) 77.46 (6.99) 78.00 (6.90)	0.46 (< 0.001)*
Gender Female Male	115 (75.20) 38 (24.80)	74.18 (7.72) 73.16 (9.13)	0.05 (0.502)**
Marital status Single Married Divorced	55 (35.90) 96 (62.70) 2 (1.30)	71.14 (7.10) 75.35 (8.23) 82.27 (0.56)	0.27 (0.001)**
Academic Degree BS MS	125(81.70) 28(18.30)	72.10 (7.04) 82.09 (7.41)	0.44 (< 0.001)*
Work Experiences (years) 0.5-7 8–15 16≥	73 (46.50) 58 (36.50) 22 (14.00)	70.22 (7.77) 77.00 (6.57) 78.15 (7.47)	0.43 (< 0.001)*

*Spearman Correlation

**Pearson Correlation

**Table 2:** Description and Pearson correlation matrix among cultural competence, empathy, job conflict and work engagement in critical care nurses

Variable (Min-Max)	Mean (SD)	1 r (p-value)	2 r (p-value)	3 r (p-value*	4 r (p-value)
(1) Cultural Competence (20–100)	74.05 (7.96)	1 (> 0.999)			
(2) Empathy (20–140)	83.44 (29.17)	0.50 (< 0.001)	1 (> 0.999)		
(3) Job Conflict (0–20)	11.00 (2.38)	-0.16 (0.049)	-0.17 (0.035)	1 (> 0.999)	
(4) Work Engagement (0-102)	43.69 (16.33)	0.14 (0.068)	0.08 (0.290)	-0.07 (0.366)	1 (> 0.999)

### Correlation between variables and multiple linear regression

The results of Pearson and Spearman correlation tests show there was a significant relationship between cultural competence and age (r = 0.46, p < 0.001), marital status (r = 0.27, p = 0.004), academic degree (r = 0.44, p < 0.001), and work experiences (r = 0.43, p = 0.001) (Table 1). The results of the Pearson test showed that cultural competence had a moderate correlation with empathy (r = 0.50, p < 0.001) and a poor and negative correlation with job conflict (r = -0.16, p = 0.049). There was no significant correlation between cultural competence and work engagement (r = 0.14, p = 0.068) (Table 2).

Only the demographic variables that had a significant relationship with the dependent variable were included in the regression model. There was no auto-correlation between the residuals, as indicated by the Durbin-Watson statistic of 1.68. There was no multi-collinearity between the predictor variables, as indicated by the VIF reading of 1.10–9.08. The initial analysis discovered that age, marital status, work experiences, and academic degree significantly influenced the regression model and accounted for 32.5% of the variation in cultural competence (F = 23.62, p < 0.001). In the following analysis, adding the main variables to the regression model, all predictor variables could explain 44.3% of the changes in cultural competence (F = 15.89, p < 0.001). The findings showed that academic degree (β = 0.36, p < 0.001) and empathy (β = 0.26, p < 0.001) could predict cultural competence (Table 3).

**Table 3:** Predictors of cultural competence in critical care nurses

Dependent Variable	Steps	Predictive variable	B	SE	β	t	P-value	R^2^
Cultural Competence	First Step	Age	5.02	2.24	0.42	2.23	**0.027**	0.390
		Marital Status	1.81	1.09	0.11	1.66	0.097	
		Work Experiences	-0.86	2.14	-0.07	-0.07	0.689	
		Academic Degree	8.82	1.35	0.42	6.51	**< 0.001**	
	Second Step	Age	4.43	2.17	0.38	2.03	0.043	0.436
		Marital Status	1.29	1.05	0.08	1.23	0.220	
		Work Experiences	-0.14	2.09	-0.13	-0.70	0.485	
		Academic Degree	7.54	1.35	0.36	5.52	**< 0.001**	
		Empathy	0.07	0.02	0.26	3.50	**0.001**	
		Job Conflict	0.06	0.23	0.01	0.26	0.793	
		Work Engagement	0.00	0.03	0.00	-0.05	0.953	

SE = Standard Error, β = Unstandardized Coefficients

* Multiple Regression Analyses

## Discussion

In this study, CCNs’ cultural competence and job conflict level were moderate, empathy was good, and work engagement was poor. The findings showed that cultural competence is significantly related to age, marital status, academic degree, work experiences, empathy, and job conflict. Academic degree and empathy variables could predict cultural competence, and the most impact was related to an academic degree.

The present study demonstrated that CCNs have a moderate level of cultural competence. A study showed that nurses born in Anglo-Saxon countries had the highest level of cultural competence and, respectively, nurses born in European and Asian countries [18]. Cultural competence is the most basic need and necessity of nursing to develop the care of patients with various backgrounds due to the increased cultural diversity and migration of nurses worldwide [19, 45]. Since many Iranian CCNs work as specialists in other countries and their migration rate is increasing, they can be considered global nurse forces [19]. This issue highlights the importance of strategies to improve cultural competence in Iranian CCNs.

This study showed that CCNs’ empathy level was good. Other studies showed that empathy in CCNs is above average in Iran [23] and high in Turkey [46] and Jordan [24]. The study’s findings by Amiri et al. revealed that nurses’ reduced capacity for empathy might be related to cultural prejudices towards patients or a lack of adequate cultural awareness [2]. The CCNs must communicate effectively and demonstrate empathy because they care for patients in danger of dying [23].

Our study showed that empathy is significantly related to cultural competence and is considered a predictive factor among CCNs. The results of Zarei et al.‘s study indicated that cultural competence and empathy are correlated [20]. Also, some studies in South Korea showed that empathy and cultural competence in nurses are correlated, and empathy is a predictor of cultural competence [28, 47]. Thus implementing policies to improve empathy among CCNs is required to enhance cultural competence. Following this, one can use the benefits of improving cultural competence, including improving patient satisfaction, increasing patient empowerment, better clinical outcomes, and better communication [20, 21]. Empathy can sensitize the nurse to the values and culture of critical patients and provide care based on cultural competence.

We revealed that the job conflict of our participants was at a moderate level. A previous study demonstrated that job conflict among Iranian CCNs was medium to high [30]. Another study showed that 77.8% of Iranian nurses experienced moderate workplace conflicts, and 16.5% had high conflicts [35]. Due to the complexity of patient circumstances, the nature of the work, the leadership style of the nurses, and the high hazards and stress levels in the critical care units, nurses experience high interpersonal conflict [48]. High-level conflict can result in challenging behaviors in the workplace and affect the quality of care for patients with critical conditions who have sensitive situations.

The results revealed a significant negative correlation between job conflict and cultural competence. This result emphasizes reducing conflict between CCNs to promote cultural competence. Identifying the contributing causes of job conflict can help nurses and nursing managers to lessen its prevalence in the workplace because studies have shown that conflict at work predicts turnover intentions and burnout [29, 35].

The current study revealed that CCNs are poorly engaged in work. Another study showed that CCNs have moderate work engagement [49], while general nurses have high work engagement [50]. Long-term outcomes of moral distress hurt the ability of CCNs to provide proper patient care, impact their ability to perform everyday job responsibilities, and lessen their work engagement. A positive work atmosphere and supportive organization encourage work engagement in nurses [49]. Reasons such as long working hours, overload, lack of professional experience, and closeness to recurring death situations can affect the CCNs’ work engagement; workload reduction, training, and Job rotation of nursing professionals among the hospital sectors are suggested.

In addition to empathy, the academic degree was also a predictor of cultural competence. In line with these results, Mareno and Hart’s study showed that graduate nurses had higher scores on cultural knowledge than undergraduate-degree [51]. Since cultural knowledge can be achieved through the training of cultural competence in undergraduate nursing [45], it seems necessary to provide continuous opportunities to improve the cultural competence of CCNs so that they interact more with different cultures. Master nurses may be more aware of cultural competence and may have received training as part of their master’s degree curriculum, compared to undergraduate-degree nurses. Due to the importance of critical care units, employing nurses with higher education in these departments is recommended.

The results of this study can help policymakers and nursing managers design and implement more effective strategies and programs to have CCNs with high cultural competence. Additionally, a well-designed program that aims to improve the cultural competence of CCNs and is appropriate enough to represent the cultural context of Iran should be implemented. Managers need to pay attention to the low levels of work engagement among CCNs and investigate the factors that support them. This study recommends using nurses with higher education to employ in critical care units and developing and implementing an educational program to foster empathy to improve cultural competence in CCNs.

One of the strengths of this study is the exploration and measurement of the association of several concepts with cultural competence. Another strength is the multicenter study design performed on CCNs at three hospitals, which may expand the generalizability of the results.

Limitations of this study include the design regarding the use of non-probability convenience sampling. Since the data collection tools were supplemented with self-reports, respondent bias and social desirability may be increased. It was challenging to convince the participants because there were many questionnaires, and they filled them out slowly. We solved this issue with more perseverance and open communication with the research participants.

Further research is necessary to comprehend the causality of the relationships between variables. Conducting more studies on job conflicts, work engagement, and their relationship with cultural competence among CCNs is recommended. Also, using a qualitative approach to understand the lived experience of critical care nurses and cultural care would be beneficial.

## Conclusion

The results of this study demonstrated that in Iranian CCNs, the level of cultural competence and job conflict were moderate, empathy was good, and work engagement was poor. There was a significant relationship between cultural competence, age, marital status, academic degree, work experiences, empathy, and job conflict. Academic degree and empathy variables could predict cultural competence, and the most impact was related to an academic degree.

## Data Availability

The datasets generated and analyzed during the current study are not publicly available due to privacy protection and ethical considerations but are available from the corresponding author upon reasonable request.

## Abbreviations

CCNs: Critical Care Nurses
ICU: Intensive Care Unit
CCU: Coronary Care Unit
ICC: Intra-class Correlation Coefficient
SD: Standard Deviation
VIF: Variance Inflation Factor
